# Healthcare professionals' acceptance of COVID‐19 vaccination for their children: A cross‐sectional study at a tertiary care hospital in Western India

**DOI:** 10.1002/hsr2.1821

**Published:** 2024-01-18

**Authors:** Gladson Vaghela, Apexa Shukla, Darshan J Dave, Aashish Lamichhane

**Affiliations:** ^1^ Gujarat Medical Education & Research Society (GMERS) Medical College Gandhinagar Gujarat India; ^2^ Department of Pharmacology Gujarat Medical Education & Research Society (GMERS) Medical College Gandhinagar Gujarat India; ^3^ College of Medical Sciences Bharatpur Nepal

**Keywords:** COVID‐19 vaccines, health personnel, knowledge, perception, vaccination hesitancy

## Abstract

**Background and Aims:**

Establishing a transparent and forthright dialog between healthcare professionals (HCPs) and the public is paramount in promoting the effective acceptance of COVID‐19 vaccination for children. Hence, this study aimed to assess the COVID‐19 vaccine acceptance, knowledge, and perception among HCPs for their children.

**Methods:**

A cross‐sectional study was conducted among HCPs at a tertiary care teaching hospital in Western India. A structured questionnaire was designed and validated to collect data. Descriptive statistics were used for data analysis.

**Results:**

The study found that more than 80% of HCPs had good knowledge about COVID‐19 vaccines, and 92.74% of them were willing to accept the vaccine for their children. Doctors were more likely to vaccinate their children, and 73% of HCPs had a favorable attitude toward immunizing their children if a new COVID‐19 vaccine was available. Academic/peer‐reviewed publications were considered the most reliable source of information for COVID‐19 vaccination, followed by government sources.

**Conclusion:**

This study found that parental vaccine hesitancy was significantly lower among the HCP group. The majority of HCPs were in favor of vaccinating their children against COVID‐19, indicating that they can serve as an effective channel for promoting parental acceptance of COVID‐19 vaccines in the community.

## Highlights

- More than 80% of healthcare professionals (HCPs) had good knowledge about COVID‐19 vaccines, and 92.74% of them were willing to accept the vaccine for their children.
- Doctors were more likely to vaccinate their children, and 73% of HCPs had a favorable attitude toward immunizing their children if a new COVID‐19 vaccine was available.
- The majority of HCPs considered academic/peer‐reviewed publications as the most reliable source of information for COVID‐19 vaccination, followed by government sources.
- The study found that parental vaccine hesitancy was significantly lower among the HCP group, indicating that they can serve as an effective channel for promoting parental acceptance of COVID‐19 vaccines in the community.

## 1 INTRODUCTION

Vaccine hesitancy has been identified as one of the top 10 threats to global health by the World Health Organization. The vaccines against COVID‐19 are the fastest vaccines ever produced for a disease^1^ and hence are a matter of concern among the population.^2^, ^3^ An increasing number of vaccines are being authorized for use in children across the globe.^4^ On January 3, 2022, the Government of India extended the COVID‐19 vaccination for children aged 15–18 years.^5^ Vaccinating children against COVID‐19 protects children from rare but severe cases of COVID‐19 infection and reduces the further spread of the virus within the community.^6^ Despite the low risk of severe illness and mortality from COVID‐19 among children, it is notable that the emergence of new variants has been correlated with an augmented prevalence of cases as well as more severe clinical manifestations in younger age groups.^7^ According to extant reports, the incidence rate of the Omicron variant among individuals aged less than 5 years was observed to be six to eight times higher in comparison to the Delta variant in the United States.^8^, ^9^ Consequently, children who have contracted the virus may be susceptible to severe pathological states such as croup and multisystem inflammatory syndrome necessitating rigorous therapeutic interventions, hospitalization, and even admission to intensive care units.^10^, ^11^ Furthermore, these individuals may endure prolonged symptoms with subsequent deleterious impacts on their overall well‐being.^12^

Although rare, it must be acknowledged that COVID‐19 fatalities can occur in children. UNICEF reports, as of January 2023, that out of the 367 million COVID‐19 cases reported from 105 countries (56% of the total global cases identified by Johns Hopkins University), 21% (75.3 million) of these cases occurred in children and adolescents aged under 20 years.^13^ For comparative purposes, the population of individuals under 20 years of age makes up 33% (2.1 billion) of the total population (6.5 billion) in the aforementioned 105 countries. Consequently, COVID‐19 vaccination remains one of the most effective measures for shielding individuals against infection. Additionally, achieving comprehensive vaccination coverage among the younger population is imperative for attaining herd immunity and ultimately subduing the pandemic.^14^, ^15^, ^16^

Although vaccinating children for COVID‐19 is of paramount importance, the issue of vaccine hesitancy among parents represents a significant obstacle. Investigations conducted in multiple countries have revealed a pervasive lack of interest among parents with regard to vaccinating their children against COVID‐19.^16^, ^17^, ^18^ In India, a study conducted by Padhi et al. indicates that merely 33.5% of Indian parents were willing to vaccinate their children against the disease.^19^ Parental reservations surrounding their child's COVID‐19 vaccination are exacerbated by the deluge of information available on social media platforms concerning the efficacy and safety of the vaccines.^20^ Therefore, establishing a transparent and forthright dialog between healthcare professionals (HCPs) and the public is paramount in promoting the effective acceptance of COVID‐19 vaccination for children.

It is therefore of the utmost importance to gain insight into the acceptance of COVID‐19 vaccination by HCPs for their children. If HCPs hold reservations and display inadequate confidence in vaccinating their own children against COVID‐19, they may encounter significant challenges in fostering trust in the efficacy and safety of COVID‐19 vaccines among the wider population, particularly among the parents whom they serve in clinical or community settings. Hence, this study aims to evaluate HCPs' knowledge, perception, and acceptability of COVID‐19 vaccination for their children.

## 2 METHODS

### 2.1 Sample and procedure

This cross‐sectional study was conducted between January and October 2022 at a tertiary care teaching hospital located in Western India. The total HCPs in the institute is 570, inclusive of doctors, nurses, pharmacists, physiotherapists, and lab technicians. We employed a purposive sampling technique to ensure a representative sample of HCWs from different departments and positions within the hospital. The study obtained prior approval from the Central Research Committee and Institutional Ethics Committee (No. GMERS/MCG/IEC/08/2022, Dated 30/04/2022) before its implementation. A sample size of 422 participants was determined using a single population proportion formula, considering a 95% confidence interval, a 5% margin of error, a 50% proportion of vaccine acceptability, and an additional 10% nonresponse rate. HCPs with children under the age of 15 were eligible for inclusion in the study, with no restrictions based on age, sex, ethnicity, religion, health status, or other factors. The study adhered to the Declaration of Helsinki.

### 2.2 Development of the questionnaire

The study utilized a multi‐item online questionnaire based on the findings of the literature review. Published COVID‐19 vaccine hesitancy questions were adopted and adapted to the study's context from validated sources.^16^, ^18^ This approach aimed to enhance the data quality and validity and enable easy comparison with other populations. The questionnaire underwent face validity by two experts, after which the questions that received aacceptability of 90%–100% of agreement were retained in the questionnaire. Later on, the questionnaire was sent to three experts to assess and each question required rating on a Likert scale. The questionnaire was also subject to a pilot study on 30 participants after which the Cronbach alpha of each question was calculated and all questions with an alpha rating above 0.70 were included in the final version of the questionnaire.

### 2.3 Questions and scale

The questionnaire was prepared in English and it comprises a total of 28 questions: 11 items on demographic details, 6 items on knowledge criteria, 5 items on perception‐related criteria, and 6 items on acceptability‐related criteria. It consisted of different types of questions, including (Yes/No) questions, open‐ended questions, and select all‐apply questions.

### 2.4 Knowledge section

The study evaluated participants' understanding of the COVID‐19 vaccine through a set of six questions, with a possible cumulative score ranging from 0 to 10. Bloom's cut‐point was utilized to categorize the overall knowledge score as “good” if it was equal to or greater than 80% (8 points).^21^

### 2.5 Perception section

The study measured participants' views on the COVID‐19 vaccine through four questions, with a possible cumulative score ranging from 0 to 7. The overall perception level was categorized based on Bloom's cutoff point, with a score of 80% or higher (at least 5.6 points) indicating a positive perception.^21^

### 2.6 Acceptability section

The study evaluated the willingness of participants to receive the COVID‐19 vaccine through a set of three questions, with a possible cumulative score ranging from 0 to 4 for each participant.

### 2.7 Data collection

In this study, data collection was conducted online through Google Forms between May 1, 2022 and June 30, 2022. Recruitment of participants was carried out through various online channels such as WhatsApp and Email, with reminders sent every 2 weeks during the study period to ensure maximum participation. Before participation, all respondents were provided with a participant information sheet (on the first page of Google Form), and given the option to skip any questions or exit the survey at any time. Written informed consent was obtained from all participants, and to ensure anonymity and confidentiality, no personal identifying information was collected. The estimated completion time for the survey was approximately 10 min. To prevent duplication of entries, a one‐attempt‐per‐device setting was implemented. No follow‐up was conducted on the participants. No compensation of any sort was provided on completion of the survey.

### 2.8 Statistical analysis

The collected data was analyzed using GraphPad Prism 9.4.1 for Windows, GraphPad Software, www.graphpad.com. Descriptive statistics were used to summarize the quantitative data, including mean and standard deviation after checking the normality of data using the Kolmogorov test. Qualitative data was presented as absolute (*N*) and relative (%) frequencies. To determine any association in knowledge groups (good vs. poor), perception (positive vs. negative), and vaccine acceptance (likely vs. unlikely) by demographic characteristics, the chi‐square test was applied. Independent sample *t* test and one‐way ANOVA analysis were used to assess any differences in mean knowledge score by demographic characteristics. The statistical significance level was set at *p*‐value < 0.05.

## 3 RESULTS

### 3.1 Demographic information

A total of 441 HCPs participated in this study and were included in the statistical analysis. A total of 441 responses were received from a total maximum pool of 570 HCPs (response rate of 77.4%). As shown in Table 1, the mean age of participants is 35.09 years (SD = 5.13, range from 21 to 52). Out of 441 participants, 51% (*n* = 227) were males and 48.5% (*n* = 214) were females. The majority of study participants were doctors (49.4%, *n* = 218), followed by nurses (29%, *n* = 128), pharmacists (14.7%, *n* = 65), physiotherapists (3.8%, *n* = 17), and lab technicians (2.9%, *n* = 13). Among all participants, most of them (68.9%) have taken a booster dose and were fully vaccinated.

**Table 1:** Demographic characteristics of healthcare professionals.

Variable		*N*	%
Total		441	100%
Gender	Male	227	51.5
	Female	214	48.5
Profession	Doctor	218	49.4
	Nurse	128	29
	Pharmacist	65	14.7
	Physio‐therapist	17	3.8
	Lab technician	13	2.9
Status of vaccination against Covid‐19	Not vaccinated	5	1.1
	1st dose taken	6	1.3
	2nd dose taken	126	28.57
	Booster dose taken	304	68.9
Number of children (0–14 years old) in household (*n* = 438)	1	219	50
	2	190	43.3
	3	24	5.47
	>3	5	1.1
Pre‐pandemic routine vaccines received for children	All vaccines received	325	74
	Some vaccines received	74	16.9
	No vaccines received	21	4.8
	Do not know	19	4.3

Abbreviations: %, percentage; N, number of participants.

### 3.2 Knowledge of the COVID‐19 vaccines

HCPs agreed that vaccination against COVID‐19 is necessary for children (62.1%, *n* = 271), whereas 92.1% (*n* = 407) of HCPs were aware that the COVID‐19 vaccine can be given to children (0–14 years). When asked questions regarding the number of vaccine (Covaxin) doses approved for children (12–17 years of age), 75.8% (*n* = 335) of participants answered two doses. Less than half of HCPs agreed to whether “children have a lower chance of being infected with COVID‐19?” Table 2 shows differences in study participants' knowledge based on demographics toward vaccination against COVID‐19 and there was significant association between good knowledge and profession, and gender, respectively. The average knowledge score was 6.5 (SD, ±1.98) (range: 0–10). Overall, 81.25% of the participants were categorized as having good knowledge (score > 8).

**Table 2:** Shows differences in healthcare professionals' knowledge based on demographics toward vaccination against COVID‐19 for their children.

Variable	*N* (%)	Knowledge‐response questions—12, 13, 14, *N* (%)			Knowledge‐response questions—15, 17, *N* (%)		
		Agree	Disagree	No	Yes	No	*p* Value
Total	441	218 (49.4)	136 (30.8)	87 (19.7)	269 (61)	172 (39)	
Gender							
Male	227 (51.5)	112 (49.3)	69 (30.4)	41 (20.2)	140 (61.6)	87 (38.3)	0.001*
Female	214 (48.5)	106 (49.5)	68 (31.7)	41 (19.1)	128 (59.8)	86 (40.1)	
Profession							
Doctor	218 (49.4)	100 (45.8)	70 (32.1)	48 (22.0)	126 (57.8)	92 (42.2)	0.008*
Nurse	128 (29)	67 (52.3)	41 (32.0)	20 (15.6)	78 (60.9)	50 (39.0)	
Pharmacist	65 (14.7)	32 (49.2)	17 (26.1)	16 (24.6)	45 (69.2)	20 (30.7)	
Physio‐therapist	17 (3.8)	8 (47.1)	6 (35.3)	3 (17.6)	10 (58.8)	7 (41.2)	
Lab technician	13 (2.9)	9 (69.1)	3 (23.1)	1 (7.7)	10 (76.9)	3 (23.1)	

*Note*: Difference tested with independent *t* test for Gender and ANOVA for Profession.

Q12. Do you believe that children have lower chance of being infected with COVID‐19?

Q13. Do you believe if children are infected with COVID‐19, the symptoms will be more severe than adults?

Q14. Do you believe that vaccination against COVID‐19 is necessary for children?

Q15. Are you aware that COVID‐19 vaccine can be given to children (0–14 years).

Q17. Can COVID‐19 vaccine protect us from influenza?

Abbreviations: %, percentage; N, number of participants.

* *p* value < 0.05 is statistically significant.

### 3.3 Perception toward vaccination against COVID‐19

**Figure 1:**
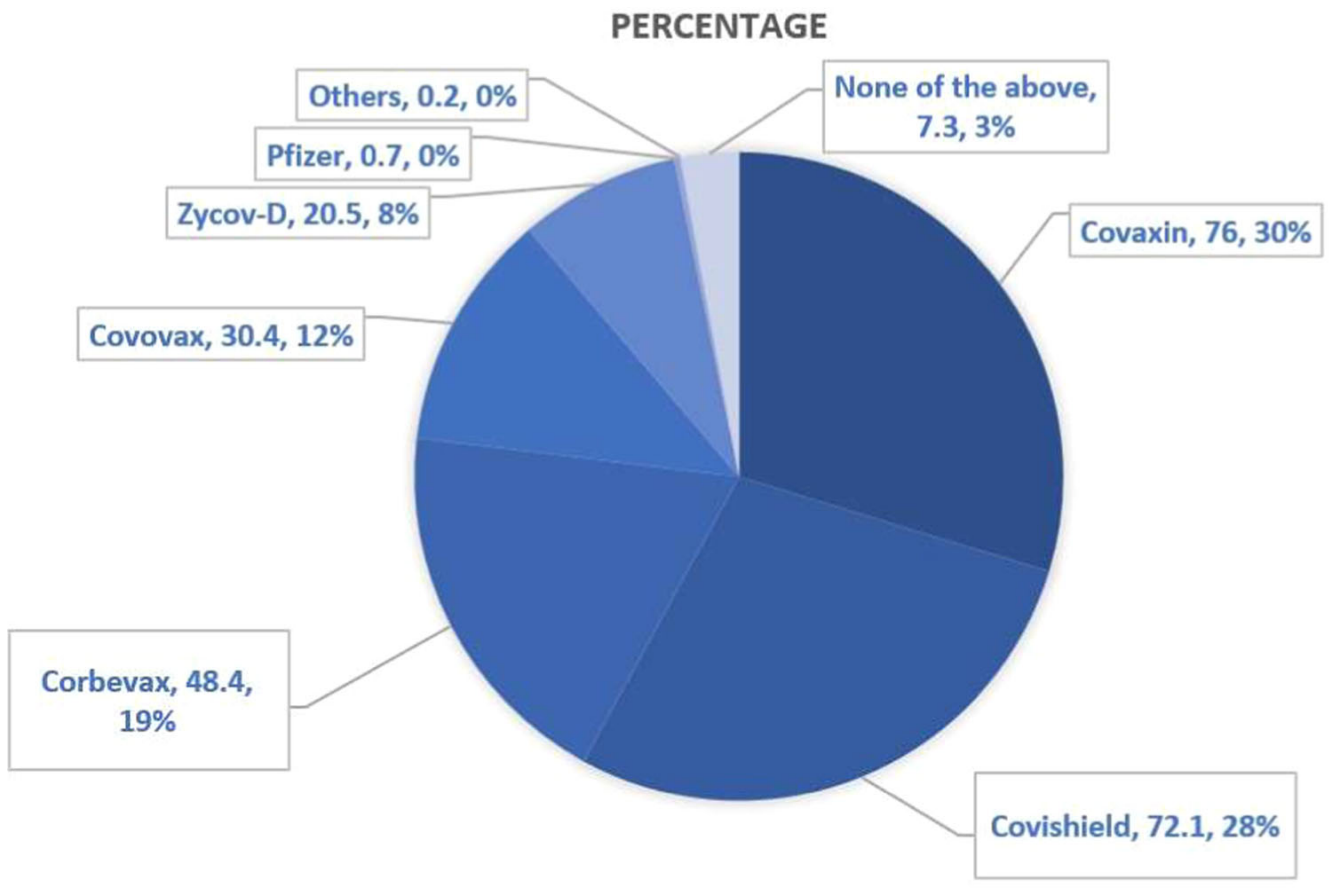
Selection of COVID‐19 vaccines by healthcare professionals.

Overall, 39.2% of HCPs were confident that COVID‐19 vaccines will be safe for children, whereas the majority of HCPs (41.7%) had a neutral viewpoint. Seventy‐three percent of HCPs are willing to accept vaccination for their children if a new COVID‐19 vaccine becomes available. The average perception score was 3.39 (SD, ±2.55) (range: 0–7). Overall, 54.55% of the participants were categorized as having a positive attitude (score > 5.6). When asked which vaccine they preferred for their child, the majority of HCPs chose to have Covaxin or Covishield followed by Corbevax as shown in Figure 1.

### 3.4 Acceptability toward COVID‐19 vaccines

**Figure 2:**
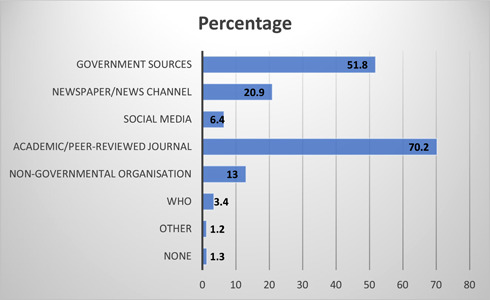
Healthcare professionals' source of information related to COVID‐19 vaccines.

The majority of the participants (76.4%) trust the existing source of information on COVID‐19 vaccination for children. The most common source of information was academic/peer‐reviewed journals (70.2%) followed by government sources (51.8%), newspaper/news channels (20.9%), and social media (6.4%) (Figure 2). More than half (53%) of the HCPs were willing to pay for vaccinating their children if the government does not provide vaccination free of charge. In addition, 26% of HCPs have already vaccinated their children, whereas 39% of HCPs will vaccinate their children after few months of vaccine approval. The COVID‐19 vaccination acceptance rate among HCPs for their children was found to be 92.74%.

**Figure 3:**
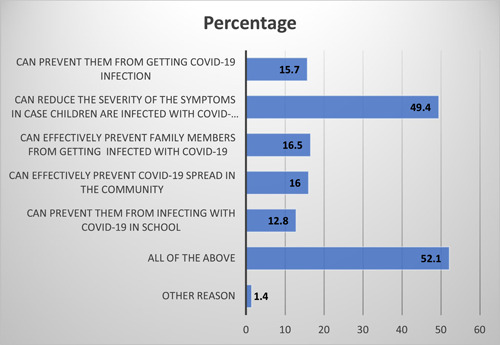
Distribution of reasons for accepting COVID‐19 vaccination by healthcare professionals.

As shown in Table 3, 80% of the HCPs were categorized as having likely vaccine acceptance. As shown in Figure 3, over 90% of HCPs expressed their reasons for vaccine acceptability, of which 52.1% of HCPs accept COVID‐19 vaccination for their children due to variety of reasons, including protection against COVID‐19 infection in school, protection against COVID‐19 infection among family members, prevention from getting COVID‐19 infection and its further spread in the community, and for the reduction of severity of infection due to COVID‐19. While 7% of HCPs have given reason for rejecting child vaccination against COVID‐19 due to uncertainty about the vaccine's safety. Table 3 shows association in HCPs' COVID‐19 vaccine acceptance for their children based on demographics, and there was no significant association found between good knowledge and gender, age group, and profession, respectively.

**Table 3:** Demographic characteristics associated with COVID‐19 vaccine acceptance among healthcare professionals for their children.

Variable	Vaccine acceptance, *N* (%)		*p* Value	OR (95% CI)
	Likely	Unlikely		
Total	353 (80.04)	88 (19.95)		
Gender				
Male	173 (76.2)	54 (23.79)	0.82*	0.92 (0.5–1.4)
Female	166 (77.57)	48 (22.43)		
Age				
≤30 years	80 (78.43)	22 (21.57)	0.78*	1.12 (0.6–1.9)
>30 years	259 (76.4)	80 (23.60)		
Profession				
Doctor	172 (78.90)	46 (21.10)	0.31*	1.28 (0.8–2.0)
Nurse	95 (74.21)	33 (25.78)		
Pharmacist	49 (75.38)	16 (24.61)		
Physio‐therapist	11 (64.7)	6 (35.2)		
Lab technician	11 (84.6)	2 (15.3)		

*Note*: Association tested using chi‐square test. *p value < 0.05 is statistically significant.

Abbreviations: %, percentage; N, number of participants.

## 4 DISCUSSION

This study represents one of the few studies conducted in India evaluating HCPs' knowledge, perception, and acceptability of the COVID‐19 vaccines for their children. We analyzed COVID‐19 vaccine acceptance levels in HCPs who play a pivotal role in vaccine awareness and education in the community, such as doctors, nurses, pharmacists, physiotherapists, and laboratory technicians. In our study, the ratio of male to female participants was found to be nearly equal. The majority of doctors who participated in our study were comprised of males (62.84%, *n* = 137), whereas nurses were predominantly comprised of females (80.4%, *n* = 103).

A study conducted by Kadam et al., to assess the HCPs'acceptability of the COVID‐19 vaccine for their children found that 90% of the participants (i.e., HCPs) were aware of the vaccine and believed in its usefulness in preventing infection (*p* = 0.008) and reducing severity (*p* = 0.009).^22^ Studies conducted by Lazarus et al. evaluated the COVID‐19 vaccine acceptance rates for 23 countries, with India having a 98.3% overall vaccine acceptance and a vaccine hesitancy of 6.3% for children among parents. This compares similarly to our study finding a 92% vaccine acceptance for children.^23^ The above study also found that India had one of the lowest rates of vaccine hesitancy both overall at 1.7% and also among children at 6.3% among the 23 developing and developed countries. This is lower than the vaccine hesitancy found for general vaccinations for pediatric population which was found to be around 60.2% in a study conducted in tertiary care hospital in Odisha by Banerjee et al.^24^

A study conducted by Miraglia del Giudice et al. on vaccine acceptance and hesitancy among parents of children found the vaccine hesitancy rate to be as high as 26.3%.^25^ The major factors correlated with the same were less educated, parents who did not get the COVID‐19 vaccine, lower concern about the severity of the disease and low perceived risk. As our study included HCPs, the average education level would be expected to be above average and the firsthand experience with COVID‐19 and the severity of the disease along with the high risk of transmission would all contribute to a lower vaccine hesitancy rate.

The safety and effectiveness of COVID‐19 vaccines for younger children is a major concern. About half of the HCPs agreed that vaccination in children is key to stop the pandemic but had a low confidence level due to concerns related to vaccine safety issues in children. The reasons for doubting the effectiveness of COVID‐19 vaccines can be attributed to several factors. One possible reason is the lack of information regarding the development and testing of the vaccines. In previous studies individuals have expressed concerns about the transparency of the vaccine development process, preferring vaccines developed in a more open and transparent manner.^26^, ^27^, ^28^ Additionally, there are concerns about whether regulatory standards were relaxed and trials rushed due to the unprecedented speed at which COVID‐19 vaccines were developed.^28^, ^29^

Despite the low level of confidence regarding the COVID‐19 vaccine's safety in children, the majority of HCPs (68.8%, *n* = 304) themselves were fully vaccinated (booster dose) against COVID‐19, either by Covishield or Covaxin, whereas more than one fourth of HCPs (28.5%, *n* = 126) got a minimum of two doses of COVID‐19 vaccine. These findings are similar to a study conducted by Sarkar et al., where 97% of HCPs completed two doses of vaccines before start of the study.^30^ In our study, we found that the majority of HCPs were willing to get their children vaccinated with either Covishield (76%, *n* = 335) or Covaxin (72.1%, *n* = 318). We found that the majority of HCPs regarded academic/peer‐reviewed journals (70.2%, *n* = 309) as their most reliable source of information, followed by government sources (51.8%, *n* = 228). This urges the importance of conducting and publishing large‐scale studies finding information about vaccine hesitancy and prevalent reasons for the same, so that effective strategies can be developed and put in place to combat the lack of information or misinformation that may be present at large.

### 4.1 Strength and limitations

An important aspect of our study is that it is one of the few studies in India carried out among HCPs who are parents. We had a small sample size to find clear statistical significance. One of the limitations of our study was being a single‐center study, underrepresentation of physiotherapists, technicians, and other miscellaneous staff, and overrepresentation of doctors. Since our participants are HCPs, if they accept COVID‐19 immunization for their children, it will have a beneficial effect on parents in their community, as their views on the acceptability of the COVID‐19 vaccine will influence parents' perspectives.

The self‐report nature of the questionnaire might also introduce social desirability bias, where participants might have reported what they perceived as socially acceptable answers, particularly given the professional standing of the participants as healthcare providers. The study also did not delve into the specifics of different vaccines and their efficacy or safety profiles, which could be a significant factor affecting vaccine acceptance.

Furthermore, the study relied on online channels for recruitment and data collection, which might have introduced selection bias. The participants' digital literacy and access to online platforms might have influenced their participation, potentially excluding a segment of the population. The anonymity of responses, while safeguarding confidentiality, also prevented any follow‐up or verification of the provided information.

Finally, the study did not account for the changing dynamics of the pandemic, including the emergence of new variants and updated guidelines from health authorities which might affect vaccine acceptance over time.

## 5 CONCLUSION

In conclusion, we found that parental vaccine hesitancy was significantly lower in our population of HCPs compared to other research studies. The majority of HCPs replied positively and were in favor of vaccinating their children against COVID‐19. The high acceptance of COVID‐19 vaccines among HCPs can serve as an effective channel for promoting parental acceptance of COVID‐19 vaccines in the community. We found that HCPs regarded academic/peer‐reviewed journals as their most reliable source of information for COVID‐19 vaccination, followed by government sources. Hence, ensuring the provision of accurate and timely information about COVID‐19 vaccines by concerned stakeholders and authorities may enhance public confidence and parental acceptance of the vaccines.

## AUTHOR CONTRIBUTIONS

**Gladson Vaghela**: Conceptualization; data curation; formal analysis; funding acquisition; investigation; methodology; project administration; visualization; writing—original draft; writing—review and editing. **Apexa Shukla**: Conceptualization; data curation; formal analysis; project administration; software; supervision; validation; visualization; writing—review and editing. **Darshan J Dave**: Methodology; resources; supervision; visualization; writing—review and editing. **Aashish Lamichhane**: Resources; software; validation; writing—review and editing.

## CONFLICT OF INTEREST STATEMENT

The authors declare no conflict of interest.

## TRANSPARENCY STATEMENT

The lead author Aashish Lamichhane affirms that this manuscript is an honest, accurate, and transparent account of the study being reported; that no important aspects of the study have been omitted; and that any discrepancies from the study as planned (and, if relevant, registered) have been explained.

## Data Availability

The data that support the findings of this study are available from the corresponding author upon reasonable request.

## References

[1] Lurie N , Saville M , Hatchett R , Halton J . Developing Covid‐19 vaccines at pandemic speed. N Engl J Med. 2020;382(21):1969‐1973. 32227757 10.1056/NEJMp2005630

[2] Sharun K , Rahman CKF , Haritha CV , Jose B , Tiwari R , Dhama K . Covid‐19 vaccine acceptance: beliefs and barriers associated with vaccination among the general population in India. J Exp Biol Agric Sci. 2020;8(Spl‐1‐SARS‐CoV‐2):S210‐S218.

[3] Rabaan AA , Al‐Ahmed SH , Sah R , et al. SARS‐CoV‐2/COVID‐19 and advances in developing potential therapeutics and vaccines to counter this emerging pandemic. Ann Clin Microbiol Antimicrob. 2020;19(1):40. 32878641 10.1186/s12941-020-00384-w PMC7464065

[4] Kasi SG , Dhir SK , Shah A , et al. Coronavirus disease 2019 (COVID‐19) vaccination for children: position statement of Indian Academy of Pediatrics Advisory Committee on Vaccination and Immunization Practices. Indian Pediatr. 2022;59(1):51‐57. 34927603 10.1007/s13312-022-2421-9 PMC8821846

[5] Ministry of Health and Family Welfare, Government of India. n.d. Accessed October 30, 2022. https://pib.gov.in/newsite/pmreleases.aspx?mincode=31

[6] Klass P , Ratner AJ . Vaccinating children against Covid‐19—the lessons of measles. N Engl J Med. 2021;384(7):589‐591. 33471977 10.1056/NEJMp2034765

[7] Torjesen I . Covid‐19: Omicron Variant is Linked to Steep Rise in Hospital Admissions of Very Young Children. 10.1136/bmj.o110 35031537

[8] Tanne JH . Covid‐19: cases in children rise sharply in US as doctors call for vaccine approval. BMJ. 2021;374:n2030. 34400412 10.1136/bmj.n2030

[9] Wang L , Berger NA , Kaelber DC , Davis PB , Volkow ND , Xu R . Incidence rates and clinical outcomes of SARS‐CoV‐2 infection with the omicron and delta variants in children younger than 5 years in the US. JAMA Pediatr. 2022;176(8):811‐813. 35363246 10.1001/jamapediatrics.2022.0945 PMC8976262

[10] Brewster RC , Parsons C , Laird‐Gion J , et al. COVID‐19–associated croup in children. Pediatrics. 2022;149(6):e2022056492. 35257175 10.1542/peds.2022-056492

[11] Levy N , Koppel JH , Kaplan O , et al. Severity and incidence of multisystem inflammatory syndrome in children during 3 SARS‐CoV‐2 pandemic waves in Israel. JAMA. 2022;327(24):2452‐2454. 35588048 10.1001/jama.2022.8025 PMC9121298

[12] Thomson H . Children with long covid. New Scientist. 2021;249:10‐11. 10.1016/S0262-4079(21)00303-1 PMC7927578 33686318

[13] United Nations International Children's Emergency Fund Data . Child Mortality and COVID‐19. DATA 2023. Accessed January 31, 2023. https://data.unicef.org/topic/child-survival/covid-19/

[14] Akarsu B , Canbay Özdemir D , Ayhan Baser D , Aksoy H , Fidancı İ , Cankurtaran M . While studies on COVID‐19 vaccine is ongoing, the public's thoughts and attitudes to the future COVID‐19 vaccine. Int J Clin Pract. 2021;75(4):e13891. 33278857 10.1111/ijcp.13891 PMC7883065

[15] Dodd RH , Pickles K , Nickel B , et al. Concerns and motivations about COVID‐19 vaccination. Lancet Infect Dis. 2021;21(2):161‐163. 33338440 10.1016/S1473-3099(20)30926-9 PMC7832277

[16] Bell S , Clarke R , Mounier‐Jack S , Walker JL , Paterson P . Parents' and guardians' views on the acceptability of a future COVID‐19 vaccine: a multi‐methods study in England. Vaccine. 2020;38(49):7789‐7798. 33109389 10.1016/j.vaccine.2020.10.027 PMC7569401

[17] Humble RM , Sell H , Dubé E , et al. Canadian parents' perceptions of COVID‐19 vaccination and intention to vaccinate their children: results from a cross‐sectional national survey. Vaccine. 2021;39(52):7669‐7676. 34688500 10.1016/j.vaccine.2021.10.002 PMC8500474

[18] Goldman RD , Yan TD , Seiler M , et al. Caregiver willingness to vaccinate their children against COVID‐19: cross sectional survey. Vaccine. 2020;38(48):7668‐7673. 33071002 10.1016/j.vaccine.2020.09.084 PMC7547568

[19] Padhi BK , Satapathy P , Rajagopal V , et al. Parents' perceptions and intention to vaccinate their children against COVID‐19: results from a cross‐sectional national survey in India. Front Med. 2022;9:806702. 10.3389/fmed.2022.806702 PMC9159272 35665354

[20] Gisondi MA , Barber R , Faust JS , et al. A deadly infodemic: social media and the power of COVID‐19 misinformation. J Med Internet Res. 2022;24(2):e35552. 35007204 10.2196/35552 PMC8812140

[21] Seid MA , Hussen MS . Knowledge and attitude towards antimicrobial resistance among final year undergraduate paramedical students at University of Gondar, Ethiopia. BMC Infect Dis. 2018;18(1):312. 29980174 10.1186/s12879-018-3199-1 PMC6035414

[22] Kadam KS , Uttarwar P . Covid‐19 vaccine hesitancy for children in parents: a cross‐sectional survey among healthcare professionals in India. Indian J Psychiatry. 2022;64(Suppl 3):S565.

[23] Lazarus JV , Wyka K , White TM , et al. A survey of COVID‐19 vaccine acceptance across 23 countries in 2022. Nat Med. 2023;29(2):366‐375. 36624316 10.1038/s41591-022-02185-4

[24] Banerjee A , Mohapatra I , Kumar A , Mishra K , Acharya G . Vaccine hesitancy: an experience from an immunization clinic of a tertiary care hospital of Eastern Odisha. J Integr Med Res. 2023;1(2):61‐64.

[25] Miraglia del Giudice G , Napoli A , Corea F , Folcarelli L , Angelillo IF . Evaluating COVID‐19 vaccine willingness and hesitancy among parents of children aged 5–11 years with chronic conditions in Italy. Vaccines. 2022;10(3):396. 35335028 10.3390/vaccines10030396 PMC8953590

[26] El‐Elimat T , AbuAlSamen MM , Almomani BA , Al‐Sawalha NA , Alali FQ . Acceptance and attitudes toward COVID‐19 vaccines: a cross‐sectional study from Jordan. PLoS ONE. 2021;16(4):e0250555. 33891660 10.1371/journal.pone.0250555 PMC8064595

[27] Pogue K , Jensen JL , Stancil CK , et al. Influences on attitudes regarding potential COVID‐19 vaccination in the United States. Vaccines. 2020;8(4):582. 33022917 10.3390/vaccines8040582 PMC7711655

[28] Tanveer S , Rowhani‐Farid A , Hong K , Jefferson T , Doshi P . Transparency of COVID‐19 vaccine trials: decisions without data. BMJ Evid Based Med. 2022;27(4):199‐205. 10.1136/bmjebm-2021-111735 34373256

[29] Shah A , Marks PW , Hahn SM . Unwavering regulatory safeguards for COVID‐19 vaccines. JAMA. 2020;324(10):931‐932. 32766736 10.1001/jama.2020.15725

[30] Sarkar P , Chandrasekaran V , Gunasekaran D , Chinnakali P . COVID‐19 vaccine hesitancy among health care worker‐parents (HCWP) in Puducherry, India and its implications on their children: a cross sectional descriptive study. Vaccine. 2022;40(40):5821‐5827. 36064669 10.1016/j.vaccine.2022.08.051 PMC9420929

